# OLDER ADULTS’ PERSPECTIVES ON ETHICAL ISSUES RELATED TO AGETECH

**DOI:** 10.1093/geroni/igad104.1037

**Published:** 2023-12-21

**Authors:** Olive Bryanton, Gerry Dragomir, Jim Mann, Marjorie Moulton

**Affiliations:** University of Prince Edward Island, Charlottetown, Prince Edward Island, Canada; AGE-WELL NCE, Toronto, Ontario, Canada; Community Engagement Advisory Network, Vancouver, British Columbia, Canada; AGE-WELL, Victoria, British Columbia, Canada

## Abstract

Most funding programs in the AgeTech sector emphasise the involvement of the end user in research and innovation activities in order to strengthen relevance, appropriateness and real-world impact. However, this engaged approach is still a work in progress and often remains a matter of tokenism with older adults. Involving older adults is seen as key when creating ethically appropriate and inclusive AgeTech for their age group. This paper based on the reflections from members of two AgeTech groups: AGE-WELL’s Older Adults and Caregivers Advisory Committee and the Research Group of Seniors 411 in Vancouver, BC. This paper highlights some ethical problems inherent in AgeTech, particularly how ageist assumptions can be built into technology-based healthcare, including the ongoing challenges of community participation, and presents a more radical agenda of older adults at the forefront of setting research priorities and shaping the development and implementation of AgeTech. Central to this is the building of community capacity; training to facilitate older adults’ engagement in the research process: mobilizing expertise within the community to address local needs; developing mechanisms for connecting older adults and researchers in an integrated knowledge mobilization process. The paper reflects on how this approach can have a significant impact on the lives of older people, and address the digital divide that marginalizes them. Remaining challenges include how to sustain older adults’ participation in innovation initiatives, and providing hard evidence to demonstrate that a more engaged approach has tangible benefits and impact in terms of developing new products and services.

